# HUMAN CENTERED DESIGN TO DEVELOP A LGBTQ+ COMMUNITY ADVISORY BOARD FOR SKILLED NURSING FACILITIES

**DOI:** 10.1093/geroni/igac059.3038

**Published:** 2022-12-20

**Authors:** Jennifer May, Jason Wheeler, Ames Simmons, Mark Hensley, Jerry O’Donnell, Joseph Wheeler, Kirby Ellis

**Affiliations:** Duke, Durham, North Carolina, United States; Duke, Durham, North Carolina, United States; Duke University, Washington, District of Columbia, United States; AARP NC, Greensboro, North Carolina, United States; Community Member, Greensboro, North Carolina, United States; Carolina Aging Alliance, Inc., Raleigh, North Carolina, United States; Liberty Healthcare, Pittsboro, North Carolina, United States

## Abstract

Lesbian, gay, bisexual, transgender, queer (LGBTQ+) adults will require inclusive skilled nursing care as they age, but there is a paucity in LGBTQ+ specific training resources for skilled nursing facility (SNF) healthcare workers. Development of community advisory boards is becoming common in research studies as funders place emphasis on stakeholder engagement, but how advisory boards are created with community member involvement at the onset remains less clear. This presentation describes the creation of a community advisory board of LGBTQ+ community members, community organizations, and SNF administrators that work together to develop LGBTQ+ resources for 25 SNFs. Advisory board members were identified using a design thinking framework and the Field Guide to Human-Centered Design. Potential board members were identified through networking with community-based organizations, existing relationships with SNFs, and by word of mouth. The advisory board consists of 3 LGBTQ+ adults, 4 SNF administrators, 1 AARP community outreach and advocacy associate director, 1 advocacy coordinator for LGBTQ+ older adults, 1 SNF physician liaison, and 1 nurse scientist. To gain buy-in, individual interviews and focus groups with potential advisory board members were conducted. Meetings provided an opportunity to describe project goals and how potential member’s expertise would guide development of resources. LGBTQ+ adult community members received compensation for their participation. The advisory board is meeting monthly and approaching the design phase. Community stakeholder involvement in early stages of development will help ensure that LGBTQ+ resources are relevant and applicable to enhance health and well-being of LGBTQ+ older adults in the future.

